# The Effects of Aerobic Exercise Training on Psychosocial Aspects of Men with Type 2 Diabetes Mellitus

**DOI:** 10.5539/gjhs.v6n2p196

**Published:** 2014-01-20

**Authors:** Mohammad Ali Sardar, Vahdat Boghrabadi, Mahdi Sohrabi, Reza Aminzadeh, Mehrdad Jalalian

**Affiliations:** 1 Department of General Courses, Faculty of Medicine, Mashhad University of Medical Sciences, Mashhad, Iran; 2 Department of Physical Education, Mashhad Branch, Islamic Azad University, Mashhad, Iran; 3 Faculty of Physical Education and Sport Sciences, Ferdowsi University of Mashhad, Mashhad, Iran; 4 Department of Physical Education, Emam Reza University, Mashhad, Iran; 5 Electronic Physician Journal, Mashhad, Iran; 6 Department of Research and Foreign Affairs, Mehrafarin Inc., Mashhad, Iran

**Keywords:** aerobic exercise training, mental health, depression, diabetes mellitus, psychosocial aspects

## Abstract

**Aim:**

This study was conducted to examine the effects of aerobic exercise training on psychosocial aspects (mental health, the aspects of physical symptoms, anxiety and insomnia, social functioning, and depression) in patients with type 2 diabetes mellitus.

**Methods:**

53 men who had type 2 diabetes mellitus for a mean duration of the disease for 3±5 years were selected purposely and classified randomly into experimental (27 patients) and a control group (26 patients). Patients in the experimental group did aerobic exercise training three times a week for eight weeks. The exercise included an aerobic activity for 45 to 60 minutes during which the patients’ heart rates were maintained at 60-70 percent of heart rate reserve on ergo meter bikes.

**Results:**

The eight-week aerobic exercise training had significant effects on mental health (p = 0.002), subscales of physical symptoms (p = 0.006), and anxiety and insomnia (p = 0.001). It had no significant effects on subscales related to disorder of social functioning (p = 0.117) and depression (p = 0.657).

**Conclusions:**

Aerobic exercise training can be considered as an appropriate program for improving the health of the patients with type 2 diabetes mellitus, and it also can improve their mental health.

## 1. Introduction

The mutual effects of mental and physical factors guarantee human health, and scientists have focused on this crucial issue for a long time. According to the definition offered by the World Health Organization (WHO, 1948), the term “health” means complete physical, mental, and social well-being and not merely the absence of disease or infirmity. Physical disease can create some effects on many aspects of human life, such as interpersonal communications, occupational performance, spiritual beliefs, and the manner of communicating. A physical disease also can cause feelings of sorrow, fear, anxiety, anger, and feelings of loneliness when it makes a person feel unsafe or become unable to handle the requirements of daily living (UK Prospective Diabetes Study (UKPDS) Group, 1998). Type 2 diabetes mellitus is a disease that has seriously endangered the health of many people. Diabetes is a metabolic disorder that causes an increase in the production of serum glucose due to the body’s inability to provide adequate quantities of insulin (ADA, 2004).

According to recent research (Larijani & Zahedi, 2002), 14 to 23 percent of Iranians over the age of 30 suffer from diabetes or impaired glucose tolerance (IGT). Up to 25 percent of those with IGT will develop diabetes in the future. Many countries, including Iran, face great danger due to diabetes (King, Ronald, & Herman, 1998). Studies have indicated that obesity and immobility have a direct relationship with type 2 diabetes mellitus (Shrestha & Ghimire, 2012).

Regular physical activities can reduce the probability of being infected with type 2 diabetes mellitus, and they also improve the control of serum glucose in diabetics (Hayes & Kriska, 2008). In 2002, the American Diabetes Association (ADA) recommended an aerobic exercise training program with the intensity of 50 to 80 percent of the maximum aerobic capacity three or four times a week for 30 to 60 minutes. Physical activities increase the basal metabolism level and improve blood circulation in all parts of the body; they also use extra calories to secret endorphins, which impart a sense of well-being (Peirce, 1999). Diabetes contributes to the occurrence if several other serious diseases, including coronary heart disease, stroke, peripheral vascular disease, and ophthalmic and renal failures (Narayan, Kanaya, & Gregg, 2003). A six percent decrease in hemoglobin A_1_c can reduce the risk of the mentioned outcomes significantly (Boule, Haddad, Kenny, Wells, & Sigal, 2001). Clear evidence indicates that physical exercise programs can decrease the HbA1c level to 0.6%. According to a study of diabetes conducted in England, a 1% decrease in the HbA1c level results in a reduction of diabetics’ risk of death, risk of myocardial infarction, and risk of micro vascular disease by 21%, 14%, and 32%, respectively (Da Costa, Dritsa, Ring, & Fitzcharles, 2004). Research results have indicated that 20 to 40% of diabetics affected by depression and physical exercises showed a positive effect on their sense of well-being, i.e., such exercises have an inverse relationship with depression. Researchers at the University of Pennsylvania found that the death rate among older diabetics who suffer from depression could be decreased by 50% within a five-year period if they participated in a depression control program (Tavella et al., 2007). Some studies have indicated that depression leads to a decrease in physical exercise, so depressed people become physically immobile and the quality of their physical health is less than that in the general population. Also, Black (1999) indicated that 31% of older diabetics suffer from depression, and most of them resort to overeating as a self-medication for depression (Black, 1999). Most studies have emphasized the positive effects of physical exercise on public health and especially on type 2 diabetes mellitus, but there still are deep gaps in our understanding of the interactions between diabetes and physical exercise (Vickers, Nies, Patten, Dierkhising, & Smith, 2006). Among these gaps is our inability to identify the appropriate methods of physical exercise that produce reliable results the patients (Gilcrest, 2004). The importance of the effects of self-control methods and non-pharmaceutical interventions on diabetics’ mental health, such as aerobic exercise training, has been demonstrated, but some contradictory research has led researchers to re-examine the issue of whether aerobic exercise training produces beneficial effects on mental health, anxiety and insomnia, socialfunctioning, and depression in patients with type 2 diabetes mellitus.

## 2. Material and Methods

The research method was a randomized controlled trial. The study population of this research was 700 patients with type 2 diabetes mellitus who had been referred to specialized diabetic clinics in Mashhad for three months. These patients volunteered to participate in this research. A screening method was developed for use in selecting participants, and the screening process involved interviews, assessment of medical records, and the types of medicines being used. Inclusion criteria were being male, having diabetes type 2 both diagnosed by specialist physician and based on medical records, age of 40-50 years old, and fasting blood glucose level ranging 150-250 mg/dl. The exclusion criteria set to be:

- Currently suffering from other chronic diseases
- Currently suffering from mental illness
- Having maintained a regular exercise program over the past 3 months
- A history of myocardial infarction (heart attack)
- Uncontrolled arrhythmias
- Third-degree heart block
- High blood pressure (over 100/200 in under treatment patients)
- Diabetes type 1
- Diabetic complications such as diabetic foot ulcers, nephropathy and microalbuminuria.

Finally, Fifty-three men with type 2 diabetes participated in this study and enrolled as previously described (Castaneda et al., 2002; Lincoln, Shepherd, Johnson, & Castaneda-Sceppa1, 2011).

The sample size was calculated based on the following formula:


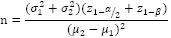


Where:

z_(1-α/2)_=1.96

z_(1-β)_= .84

σ_1_=8.7

σ_2_=8.4

μ_2_=0.3

μ_1_=0.3

n = 16

The participants were classified randomly into two groups, i.e., the control group and the experimental group using simple randomization. We used the single blind method to make sure quality of the treatment and research ethics. The two groups were matched for age, weight, past history of disease, Body fat, and HbA1c level. The research was approved by Moral Committee of Mashhad University of Medical Sciences. Before any test were conducted, the participants completed a general health questionnaire (GHQ- 28) with Chronbach’s coefficient alpha (r = 70%). The experimental group participated in aerobic exercise training course three times a week for eight weeks. Each session on the ergo meter bikes (ADA, 2004, 2010) lasted 45 to 60 minutes, on average, with the participants at 60-70% of their maximum heart rate. In addition to controlling their heart rates, we used the Borg Scale (Borg, 1982) to assess the participants’ rate of perceived exertion (RPE) for use in controlling the intensity of the aerobic exercise training, as well as active resting. The control group did not participate in any exercise activities for the eight-week period. The control group was advised to use their routine diet and daily exercise and the routine medicine as before. When the eight-week period of aerobic exercise training was completed, both groups participated in the next test. Inferential statistics, such as the t-test in the form of independent samples, i.e., the Kolmogorof-Smrirnov test about the normality of the observations and Cronbach’s Coefficient Alpha, were used to determine the reliability of the measuring tools. A significance level equivalent to p < 0.05 was used in this research.

## 3. Results

We had a 100% response rate and the Table 1 shows the Features of all the subjects in groups, e.g., age, drug treatment, diabetes history (yr) and HbA1c levels before performing the tests. The results showed no significant difference between the two groups.

**Table 1:** Characteristics of patients with type 2 diabetes in the experimental and control groups

Index	Experimental group Mean ± SD	Control group Mean ± SD	S or NS
N	27	26	-
Drug treatment before training	2(metphormin+cloropropamid)	2(metphormin+cloropropamid)	-
Age(yr)	44.93±5.35	45.56±5.41	**Ns**
Diabetes history(yr)	5.2±2.4	5.38±3.4	**Ns**
Weight (kg)	84.86±5.54	86.03±4.96	**Ns**
Fat%	29.94±6.35	31.14±6.07	**Ns**
HbA1c	7.66 ± 1.31	7.18 ± 1.01	**Ns**

S: Significant at (p < 0.05); Ns: Not significant at (p < 0.05)

The results in Table 2 show that the average difference related to mental health before testing and after testing was negative (-5.35 ± 5.52). In order to compare the difference between the pre-test and post-test mental health grades, the quantity of t was measured in the control and experimental groups and found to be 3.368. Thus, we can conclude that aerobic exercise training has a significant effect on the mental health of patients with type 2 diabetes mellitus (p = 0.002). Also, concerning the subscales of physical symptoms reported in the mental health questionnaire, the measured quantity of t to compare the difference between the pre-test and post-test grades for the two groups was 2.939. Consequently, aerobic exercise training has significant beneficial effects on the subscales of physical symptoms (p = 0.006).

**Table 2:** Grades of psychological factors for the control and experimental groups before and after training

Index	Group	Stage	Mean± SD	Mean dif± SD	t	Sig.
**Mental Health**	Control	Pre-test	39.94±4.52	0.13±2.91	3.368	(p=0.002^*^)
		Post-test	39.81±5.74			
	Experimental	Pre-test	42.12±3.03	-5.35±5.52
		Post-test	36.76±3.70	
**Physical Symptoms**	Control	Pre-test	10.13±1.96	0.19±1.68	2.939	(p=0.006)
		Post-test	9.94±2.67			
	Experimental	Pre-test	10.88±1.99	-2.12±2.05		
		Post-test	8.76±1.25			
**Anxiety and Insomnia**	Control	Pre-test	9.69±3	0.06±1.18	3.536	(p=0.001)
		Post-test	9.75±2.95			
	Experimental	Pre-test	10.94±1.81	-2 ±2.03		
		Post-test	8.94±1.24			
**Social Functioning**	Control	Pre-test	12.88±1.14	-0.19±1.51	1.619	(p=0.117)
		Post-test	12.69±1.95			
	Experimental	Pre-test	13.12±1.05	-1.35±2.52		
		Post-test	11.76±2.35			
**Depression**	Control	Pre-test	7.25±0.57	0.19±0.54	0.448	(p=0.657)
		Post-test	7.44±0.89			
	Experimental	Pre-test	7.18±0.52	0.12±0.33		
		Post-test	7.29±0.58			

Concerning the subscales of anxiety and social functioning disorder reported in the mental health questionnaire, the mean difference in the post-test grades of the experimental group was measured as a negative quantity compared to the pre-test grades (-2±2.03 and -1.35, respectively). But considering the difference in the grades between the pre-test and post-test results in both groups, the results indicated that aerobic exercise training had a significant effect on the subscales of anxiety and insomnia (p = 0.001), while its effect on the subscale of social functioning disorder was insignificant (p = 0.117). Finally, by comparing the difference in the grades related to depression between pre-test and post-test results in both groups, it can concluded that aerobic exercise training has no significant effect on the subscales of depression (p = 0.657). Table 2 shows the grades of mental health, physical symptoms, anxiety and insomnia, social functioning disorder, and depression in both groups. If the grades in the mental health index from the GHQ reached the lowest levels in the subscales of the aspects of physical symptoms, anxiety and insomnia, social functioning, and depression, the participant’s state of mind was better.

## 4. Discussion

In this study, the training program was in accordance with the predicted training program intensity and duration of aerobic exercise training recommendation made by ADA in 2002, i.e., an aerobic exercise training program should be at 50 to 80% of maximum aerobic capacity and conducted three or four times a week for 30 to 60 minutes each time (“Diagnosis and classification of diabetes mellitus,” 2004). According to the results obtained, the selected aerobic exercise training had a significant effect on the mental health of diabetics. So, the results are in accordance with the findings offered by researchers at the University of Pennsylvania (Tavella et al., 2007), Vickers et al. (2006) and Gilcrest (2004).

The selected aerobic exercise training had significant effects on the subscales of the physical symptoms of anxiety and insomnia in patients with type 2 diabetes mellitus in accordance with the findings offered by the above-mentioned researchers. However, it had no effect on the subscales of social functioning disorder and depression, which was inconsistent with the findings of the aforementioned researchers. The reasons for these different findings may be due to duration, intensity, and type of exercise training (aerobic exercise in water), because such exercise is more pleasant than other aerobic exercises that are not conducted in water.

Generally, we can conclude that physical exercises can be mentally beneficial for healthy people and people who are ill (Amsterdam, Shults, Rutherford, & Schwartz, 2006; Solanki, Dubey, & Munshi, 2009). Such physical activities have a direct relationship with mental health; quality of life, such as the sense of well-being, self-understanding, and mental health and inverse relation with anxiety, depression. Although the actual process resulting from physical activities is not clear, their value for improving mental health obviously has been proven. The advantages of physical activities in improving the mental health of diabetics may be due to the effects of aerobic exercise on the structure and biochemistry of muscles and Vo_2_max and, consequently, the positive changes (such as increasing oxidative enzymes and capillary density)(Castro-Ake, Tovar-Espinosa, & Mendoza-Cruz, 2009) that result in improving the glucose transition process and decreasing the insulin resistance level in cells(Paschalides et al., 2004). Indeed, the adaptation caused by aerobic exercise is due to the fact that the diabetics’ bodies experience an increase in the vascular density in the muscles and improved Vo_2_max and oxidative enzyme activities in their skeletal muscles. Also, aerobic exercise can increase the patient’s sensitivity to insulin so that a lower level of insulin is required to regulate the serum glucose level (W. J. Katon et al., 2004). This improvement in insulin sensitivity may be due to the insulin binding capacity of each muscular cell receptor (W. Katon et al., 2003). Also, there is a probability of increasing hepatic sensitivity. So, the patient needs lower insulin level to absorb additional insulin through blood circulation. In fact, this process is considered to be a fitness state that decreases the need for insulin during different rest times with the light and heavy intensity of different training sessions. In this situation, aerobic exercises can decrease plasma insulin levels during rest times and also reduce the production of insulin when performing glucose tolerance tests(Eren, Erdi, & Sahin, 2008; Ikeda, Aoki, Saito, Muramatsu, & Suzuki, 2003). Both processes denote insulin sensitivity and decrease the need for insulin by patients with type 2 diabetes mellitus.

One of the theories about mental-social changes related to physical activities (sedation) may be the activation of the central nervous system and the secretion of endorphins (Rakovac et al., 2004). Peirc argued that physical exercise can increase basal metabolism and improve blood circulation in all parts of body and also use extra calories, as well as promoting a sense of well-being by secreting endorphins (Peirce, 1999). Vickers argued that diabetics usually suffer from depression, which prevents them participating in physical activities. Hence, it seems that they need more prolonged physical activities, and the types of such exercises must be selected only by themselves. Obviously, it would be better to add more pleasant physical activities to their training programs, such as water sports (Vickers et al., 2006).

## 5. Conclusions

The results of this research indicated that regular aerobic exercise training can improve the general health of the patients with type 2 diabetes mellitus and that it also plays an important role in decreasing the anxiety level that results from their diabetes. It also controls their insomnia. All of these mental changes can improve the health of diabetics physiologically and also help them control their disease.
